# Responding to Covid-19: an analysis of position statements of gerontological societies worldwide

**DOI:** 10.1007/s10433-022-00700-7

**Published:** 2022-04-18

**Authors:** Caroline Emmer De Albuquerque Green, Thomas Scharf, Eva-Marie Kessler

**Affiliations:** 1 NIHR Health and Social Care Workforce Research Unit, ARC South London, The Policy Institute at King’s, King’s College London, London, UK; 2 Health Sciences Institute, Newcastle University, Newcastle upon Tyne, UK; 3 Department of Psychology, MSB Medical School Berlin, Berlin, Germany

**Keywords:** Social gerontology, Research, Equality and inclusion, Long-term care

## Abstract

The Covid-19 pandemic, with its adverse implications for older adults, has generated unprecedented public interest in issues around age and ageing globally. We systematically investigated the responses of national gerontological and geriatric societies (NGGS) to emerging challenges during the first wave of the pandemic. Framed within traditional research topics in gerontology, the aim was to identify the spectrum of focal points and positions directed towards governments, policy makers, researchers and society. A comprehensive, two-phased data collection strategy generated *N* = 22 position statements of NGGS affiliated to the International Association of Gerontology and Geriatrics. Using Ayalon et al. (J Gerontol Ser B, 2020. 10.1093/geronb/gbaa066) thematic categorisation of gerontological research, we applied quantitative and qualitative content analysis to analyse “calls for action” within the statements. The content of NGGS’ position statements show a high level of agreement on the salient topics during the first wave of the pandemic and reveal shared values such as equality, diversity and inclusion of older adults and the discipline of gerontology to be an applied one with relevance to policy and practice. The results can support future interdisciplinary research in gerontology post Covid-19 based on a vision to contribute to a society of all ages.

**Supplementary Information:**

The online version contains supplementary material available at 10.1007/s10433-022-00700-7.

As Covid-19 spread during 2020, older adults worldwide disproportionately experienced greater adverse effects from the pandemic including higher risk of infection, more severe complications and increased mortality. Global policy responses to the risk factor of “older age” in the pandemic generated unprecedented public interest in ageing issues. Internationally, important questions emerged concerning the appropriateness of policy measures based on application of chronological age, age-based discrimination in access to health and social care, and measures aimed at protecting care-home residents. Within this context, gerontologists felt challenged to position themselves in relation both to the pandemic and associated societal and political responses.

This study systematically investigates the content of position statements of national gerontological and geriatric societies (NGGS) affiliated to the International Association of Gerontology and Geriatrics (IAGG) published in response to the pandemic. While NGGS produced numerous documents and resources, including guidance and recommendations related to Covid-19, blog posts and academic articles, we focus on position statements as characteristically representative of NGGS’ distinct standpoints at this moment of time. Our analysis was guided by Ayalon et al. (2020) analysis of gerontological research communities’ traditional areas of interest, with their analytical framework being used to identify common and opposing themes in position statements with a view to informing future interdisciplinary research in gerontology.

## The role of national gerontological and geriatric societies in defining research priorities

Gerontology has been defined as “the comprehensive multidisciplinary study of aging and older adults” (Bass and Ferraro 2000) and is typically distinguished from geriatrics, the study of the health and care of older adults. As a field, gerontology has been lauded for producing a distinct and unique body of research oriented towards influencing policy agendas and practice (Samanta 2017). In 2005, the Gerontological Society of America recognised gerontology as a stand-alone discipline. However, debate continues over whether gerontology exists as a discipline in its own right or as an addendum to such established disciplines as biology, sociology and psychology (Ayalon et al. 2020; Bass 2013). Alkema and Alley (2006) previously argued that to strengthen its status as a discipline, gerontology must identify itself as interdisciplinary rather than multidisciplinary, with its own intellectual purpose, a unique contribution beyond those from the source disciplines, a common language and shared values.

To inform gerontology as a discipline, Ayalon et al. (2020) thematically classified titles of papers published in eight international psychological and social gerontology journals between 1961 and February 2020. Using natural language processing, they identified 36 thematic clusters (henceforth “categories”) and ranked them according to their frequency of appearance among all publications. They reported a trend towards a “biomedicalisation” of the field, i.e. a focus on themes such as cognitive ageing, long-term care and health. Psychological and social aspects of ageing, including the topics of morals and ethics or suicide and nutrition, received less attention, at least by scholars publishing in these journals (Ayalon et al. 2020, p. 5).

As representatives of gerontology, a central purpose of gerontological societies is to support research, education and training in relation to ageing and older adults. The IAGG is an umbrella organisation of around 73 NGGS located in 65 countries across five world regions. IAGG member societies typically seek to promote achievement in gerontological research and training, with activities being pursued “with a view of enhancing the highest quality of life and well-being of all people as they experience aging at individual and societal levels” (IAGG n.d.). During the pandemic, many IAGG member societies adopted multiple purposes beyond their traditional focus on research and education, including acting as information and resource hubs for the general public and care professionals, and campaigning and advocating by publishing position statements. Such statements seek to communicate certain points of view on particular topics of interest and demand change or action from a range of people and/or organisations concerning the topic/s of interest. Using Ayalon et al.’s framework, statements point to priority areas identified by NGGS during the pandemic’s first wave, which can be inferred as defining shared values and interests. These values may inform future international, interdisciplinary gerontological research collaborations and strengthen gerontology as a discipline.

## The present study

The Covid-19 pandemic and its wide-ranging impacts on older adults pose complex challenges for society, policy makers and researchers. It thereby offers a pressing opportunity for gerontology as a discipline to contribute knowledge and insights through research that can help to resolve the pandemic and mitigate its consequences for individuals and societies. Against this background, our main research question was: What priority areas for potential research did NGGS (i.e. organisations promoting gerontological research, education and training) identify during the first wave of Covid-19 infections? Specifically,

1. How many (and what proportion of) NGGS published statements on Covid-19 within this time period (December 2019 to September 2020)?
2. Drawing on Ayalon and colleagues’ categories of traditional research areas of gerontology, which topics were considered in published statements? Which categories were best and least well represented in statements?
3. (Within the respective categories:) What are the societies’ positions, and what are common and opposing positions? How do such positions vary between countries?

To answer these questions, we used consecutive steps of quantitative and qualitative content analysis to identify the spectrum of focal points and positions directed towards governments, politicians, policy makers, researchers and society at large.

## Methods

### Data collection

#### Inclusion criteria

We collected and analysed NGGS’ position statements, regardless of language of publication, using four inclusion criteria:

1. Originated directly from a NGGS affiliated to the IAGG;
2. Published between December 2019 and September 2020;
3. Addressed issues around Covid-19 and older adults;
4. “Outward-facing” and change oriented, i.e. published for a general readership addressing specific topics and demanding change, for example in policies, behaviour and/or language. This was determined by location of publication (e.g. public websites rather than member-only website areas), references within documents to target groups (e.g. government, politicians, journalists), and key words in the text (modal verbs, such as “must”, “need”, “should”, “oblige”).

Excluded from the sample, with a few exceptions, were: blog posts discussing Covid-19, videos, podcasts, “inward-facing statements” (i.e. statements published in member-only newsletters or website areas thus restricting readership), recommendations, letters to government departments outlining a stance on a particular topic without any calls for action, journal editors’ statements, and media statements.

#### Procedure

Data collection was undertaken between 15 June and 15 September 2020 by the lead author in discussion with co-authors. First, the lead author gathered contact details of national member societies of the IAGG, including links to societies’ websites and social media tabs as well as names of societies’ presidents and administrative leads. Second, each society’s websites, twitter feeds, Facebook/Instagram and LinkedIn pages (if available) were searched for statements that potentially met the inclusion criteria. Some NGGS’ websites and/or documents were available in the national language(s) only. In these cases, an online translation platform was used to identify potential documents meeting the inclusion criteria. The project team discussed documents for inclusion or exclusion. Third, around 50 NGGS were contacted by e-mail or social media. The research was outlined and each society was invited to submit any documents deemed by the society’s representatives to meet the inclusion criteria. This third step was intended to aid transparency around the project and fill possible gaps in step two of the data collection process. NGGS were invited to submit responses by a specified deadline. Around one-third of societies responded, with frequent references to texts published on societies’ websites.

#### Data overview

After considering documents identified during data collection or received per invitation, 22 statements were deemed to meet all inclusion criteria. Twenty of these statements were published across 13 NGGS (Argentina (SAGG) = 2; Australia (AAG) = 2; Austria (ÖGGG) = 1; Brazil (SBGG) = 1; Canada (CAG) = 1; Czech Republic (CGGS) = 1; Finland (FGS) = 1; France (SFGG) = 1; Germany (DGGG) = 4; Ireland (IGS) = 3; Portugal (SPGG) = 1; Spain (SEGG) = 2; UK (BSG) = 1). Two further statements were shared across NGGS; one instigated by France and endorsed by 31 NGGS in Europe, Asia, Africa and Latin America among other organisations, the other a statement by 14 of the Committee of Latin America (COMLAT) countries of the IAGG. We used a professional service to translate 11 statements from their original language into English. The sample is further described in the findings section. Table 1 presents the 22 statements with reference to national society of origin, title and publication date. All statements (original/translation) are available here: https://tinyurl.com/jjdutr69.

**Table 1:** Overview of categories and their prominence within the NGGS’ position statement

Prominence of category in study’s sample	Ranking per number of statements coded/Ayalon et al.’s (2020) ranking	Category	Number of statements that referred to the category at least once within the “calls for action” *N* =	Statements’ countries of origin with at least one reference to the category
1	1/2	Long-term care and formal care (see various sub-categories in this table for ranking: Healthcare/social care/institutional care/care at home/care in the community/care in hospital	19	Argentina; Australia; Austria; Brazil, Canada; Latin America; Czech Republic; Finland; France; Germany; Ireland; Portugal; Spain; UK
2	2/8	Interventions	16	Argentina; Austria; Brazil; Canada, Czech Republic; Finland; Germany; Ireland; Portugal; Spain; Australia; France; UK
3	3/14	Social relations	15	Argentina; Australia; Canada; Czech Republic; Finland; Germany; Ireland; Portugal; Spain; France; UK
4	4/15	Communication (and messaging)	14	Argentina; Australia; Austria; Canada; Czech Republic; Germany; Ireland; Portugal; UK
5	5/34	Moral, ethics and human rights (subsection of moral and ethics)	13	Argentina; Australia; Czech Republic; Finland; Germany; Ireland; International; Portugal; Australia; France; UK
6	6/18	Care workers	13	Argentina; Australia; Austria; Canada; Czech Republic; Germany; Ireland; Portugal; Spain; UK
7	7/21	Policy	13	Argentina; Australia; Canada; Finland; Germany; Ireland; Portugal; Spain; UK
8	8/22	Ageism	13	Argentina; Austria; Canada; Czech Republic; Germany; Ireland; International; France; Portugal; UK
9	NA	Healthcare	13	Argentina; Australia; Austria; Brazil; Regional; Finland; Germany; Portugal; Spain; France; UK
10	NA	Institutional care	12	Argentina; Australia; Canada; Finland; Germany; Ireland; Portugal; Spain; France; UK
11	9/4	Health	12	Argentina; Austria; Canada; Regional; Czech Republic; Finland; Germany; Portugal; Spain; France; UK
12	10/31	Frailty and vulnerability	10	Argentina; Austria; Canada; Czech Republic; Finland; Ireland; Portugal; Spain; France
13	NA	Social care	9	Argentina; Austria; Finland; Germany; Ireland; Portugal
	11/12	Community	9	Canada; Czech Republic; Germany; Ireland; Portugal; Spain; Australia; France; UK
14	12/24	Geriatrics and gerontology	8	Brazil; Canada; Germany; Portugal; Australia; France; UK
15	13/5	Family and informal care	8	Argentina; Austria; Czech Republic; Germany; Ireland; Portugal; UK
16	NA	Care at home	8	Argentina; Austria; Finland; Germany; Ireland; Portugal
17	NA	Care at hospital	8	Argentina; Australia; Austria; Finland; Germany; Portugal; Spain; France
18	14/16	Successful aging	7	Argentina; Germany; Ireland; Portugal; UK
19	15/20	Economic status and poverty	7	Canada; Finland; Germany; Portugal; UK
20	16/3	Emotionality and personality	7	Australia; Austria; Canada; Czech Republic; Germany; Portugal; UK
21	18/6	Physical functioning	6	Australia; Argentina; Finland; Germany; Portugal; UK
22	19/23	Death	6	Brazil; Regional; Finland; Portugal; Spain; UK
23	19/7	Mental health	5	Austria; Canada; Germany; Ireland; UK
24	20/33	Intergenerational	5	Germany; Ireland; Portugal; Australia; UK
25	NA	Care settings in the community	5	Austria; Germany; Ireland; Portugal; France;
26	21/28	Technology	4	Germany; Ireland; Portugal; UK
27	22/30	Elder abuse	4	Australia; Argentina; Canada; Portugal
28	23/1	Cognitive functioning	3	Austria; Canada; Germany
29	24/9	Countries and nations	3	Brazil; Canada; Portugal
30	25/35	Volunteering	3	Czech Republic; Germany; Portugal
31	26/27	Longevity and morality	2	UK; Portugal
32	27/13	Work and retirement	2	Germany; Portugal
33	28/17	Ethnicity and minorities	2	Germany; Canada
34	29/26	Life span	2	Ireland; Portugal
35	30/29	Religion and spirituality	0	
36	31/10	United States	0	
37	32/11	Gender and sex	0	
38	33/19	Marital relationships	0	
39	34/25	Rural and urban	0	
40	35/32	Immigrants	0	
41	36/36	Sleep	0	
42	37/37	Addictions	0	
43	38/38	Suicide	0	
44	39/39	Nutrition	0	

### Coding and analysis

Using analysis software (NVIVO), coding involved three systematic steps and three researchers, i.e. first author, senior author, independent researcher. In step one, the first and senior author independently identified text passages representing “calls for action” (CFA) (rather than “background” passages), as they most succinctly represent the statements’ content. A CFA passage was characterised by including modal verbs (e.g. must, should, need to, are obliged to, require) expressing a request, obligation, suggestion, order or advice. In contrast, background passages provided explanations, facts, theory or information, usually pre-empting or reasoning for CFA. Agreement between the two coders on the categorisation of material was almost perfect (98%).

In step two, quantitative content analysis (White and Marsh 2006) was undertaken to determine the frequency of categories and to prepare for subsequent qualitative coding. To do so, we referred to Ayalon et al. (2020) initial coding framework, which identified 39 categories of gerontological research. Using five statements from the sample, the coding framework was pre-tested by coding CFA passages (as identified in step one) using these 39 categories. The test-phase was undertaken by the lead and senior authors, who subsequently developed a common coding manual and systematic decision rules for each category. The initial coding framework was extended by two additional categories (i.e. communication and messaging; unclassified). Three categories were divided into further sub-categories, which we felt were insufficiently represented for the purposes of this research by the categories of Ayalon et al. These were: (1) “long-term care and formal care” with the sub-categories of institutional care; care at home; hospital care; care in the community; health care; social care; (2) “ageism” with the sub-category of heterogeneity and diversity of older people; and (3) “moral and ethics” with the sub-category of human rights. Using the tested and extended coding framework, manual and rules (Supplementary Material A1), data from the entire sample were coded by the lead author. To check reliability of the lead author’s coding, an independent researcher coded five statements (i.e. 22% of the sample). With inter-rater agreement being highly satisfactory (i.e. 85%), the lead author’s coding was accepted. After this step, common and less common topics of interest emerged from the statements as quantified by the number of statements coded per category in the coding framework.

In step three, the CFA content within each main and sub-category in the coding framework was inductively analysed for common themes (summative qualitative content analysis, Schreier, 2012). Data-driven thematic categories arising from this process were subsequently used to interpret the data and report commonalities and differences between statements. Co-authors collaborated in the analytical process through frequent discussion of emerging themes. Supplementary material includes an example of the coding under steps 2 and 3 using one of the statements and an illustration of step 3 using one of the codes (Supplementary Material A2/A3).

## Results

### Sample

The 22 position statements were published by NGGS globally, with most originating in Europe (*N* = 15, 68%), four in Latin America (*N* = 4, 18%) and three (*N* = 3, 14%) in Australia and Canada. No position statements directly published by NGGS in Asia or Africa were located. As such, the proportion of NGGS affiliated to the IAGG with at least one statement is 18%. When taking into account the international position statement and follow-on global “Old Lives Matter” campaign, aimed at combatting ageism and raising awareness of age-based discrimination, and its endorsement by various Asian, African and Eastern European NGGS, the proportion of NGGS with a statement rises to 67%.

**Fig. 1:**
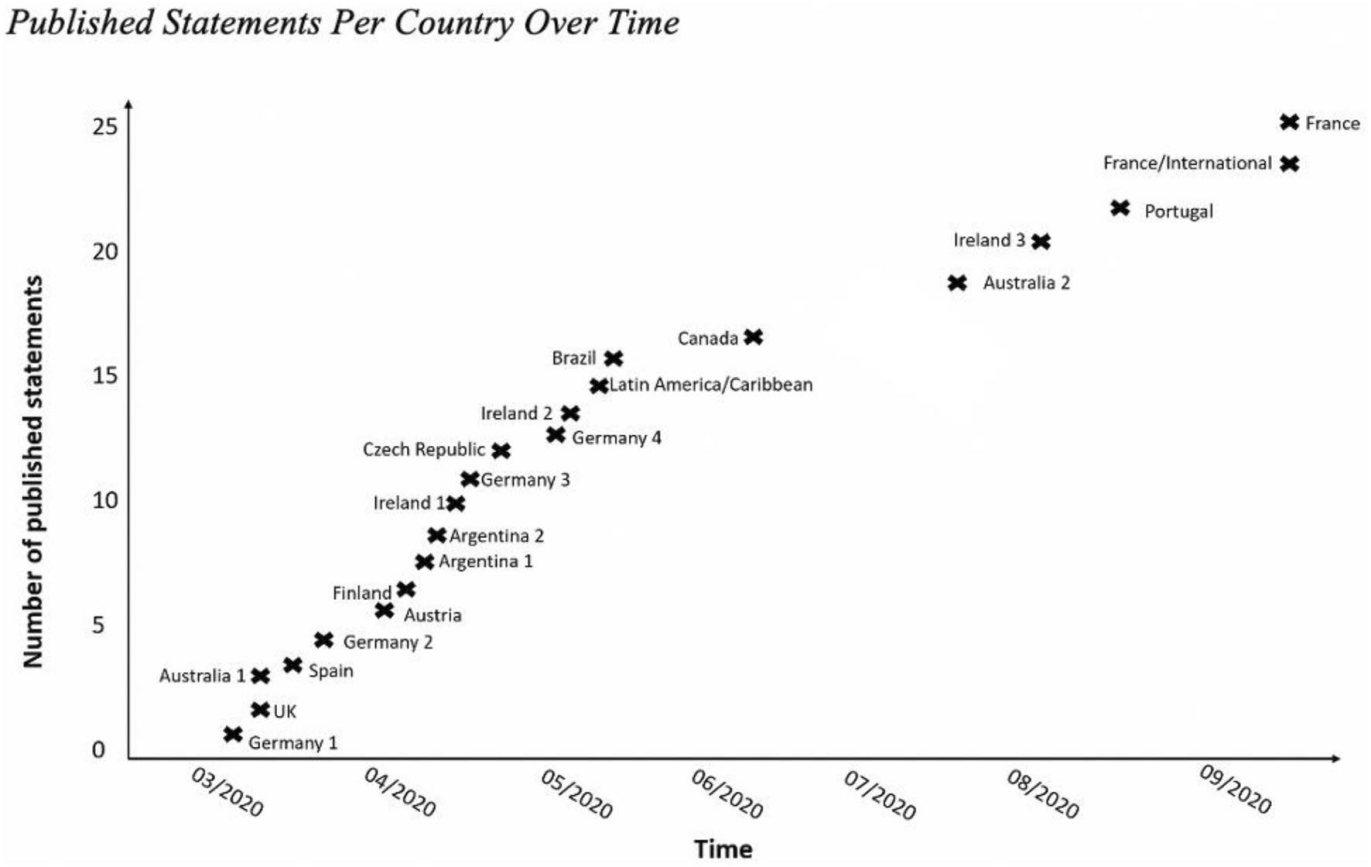
Published statements per country over time

The date of publication varied, with most statements (*N* = 15; 68%) published between March and June 2020 (Fig. 1). No data was collected that explains why NGGS decided to publish at a specific point of time or multiple statements.

### Quantitative results

Table 1 presents an overview of the prominence of categories in the study’s sample as well as the number of statements and the NGGS that referred to the category at least once within their various CFA. The table compares the ranking to that of Ayalon et al. (2020) categories. Accordingly, the top five categories identified by Ayalon et al. and this study differ significantly with the exception of “long-term and formal care”, which ranked second in both studies. In comparison, the top category of Ayalon et al.—cognitive functioning—ranked twentieth in this study, whilst social relations and interventions were more prominent in this study, together with the new (sub-)categories of human rights and communications and messaging. There was greater agreement between the two studies regarding the 10 least represented categories, with the notable exception of “moral, ethics and human rights”, which was in this study’s top five and in the bottom 10 of Ayalon et al. (2020). Accordingly, the categories of nutrition, suicide, addiction, sleep and immigrants remained some of the least represented topics in this study.

### Qualitative results

We now report results from the qualitative content analysis of the most common categories amongst position statements, highlighting common themes and differences among the NGGS’ approaches as reflected in their CFA. Results for less common categories, with supporting quotations for each category, are available as supplementary material (Supplementary Material A4).

### Long-term care and formal care

Overall, this represented the most prominent category.

#### Institutional care

Institutional care, referring to care of older adults in residential homes, was a major topic of interest for most NGGS. Societies recognised that residential homes, as settings where people faced higher risks of severe or lethal Covid-19 infections, could and then did become hotspots in the pandemic and therefore required special concern and support. Furthermore, several NGGS (e.g. Australia, Argentina, Germany) expressed concern about the treatment of care-home residents during the pandemic, demanding that residents’ basic human and/or constitutional rights, including the right to social participation (Germany), liberty (Germany) and health (Australia), were guaranteed.

Regarding infection control and response, there was consensus that protective measures and action protocols must be implemented. However, statements indicated some opposing views, especially in relation to isolating and treating care-home residents with Covid-19 infections in care homes. While one society (Germany) rejected blanket isolation measures for care-home residents out of concern over the proportionality of such a response in relation to residents’ right to liberty, another (Spain) proposed the total isolation of residents to avoid spreading the virus.

Some statements also indicated potential disagreement over the issue of hospital care versus care in residents’ care homes during or following a Covid-19 infection. While one society (Portugal) argued that care homes must not replace hospitals as the pandemic unfolded, others (Spain, Austria) advocated for care in the care home if possible, citing as a justification the need to relieve hospitals. Various national societies, including in France and Canada, argued for improved quality of care and living conditions inside care homes post-pandemic. The NGGS in Ireland demanded increased attention on developing alternatives to institutional care for older adults.

#### Healthcare

Various NGGS (Austria, COMLAT) demanded that geriatric patients with Covid-19 infections continue to receive individualised healthcare from initial infection to the rehabilitative or end-of-life phase. Associated CFA rejected blanket triage based on chronological age, the neglect of palliative or rehabilitative care standards to the detriment of patients’ wishes and needs. Some NGGS (Portugal, Australia) argued for equal access to healthcare, specifically for care-home residents. Others (Germany, Austria, Portugal) called for telecare to be provided by phone, video or other media. One society’s statement (Brazil) stood out, focusing solely on treatment of older adults with Covid-19 infections outside the remit of ethically approved research studies. The NGGS in Brazil demanded that older adults should not receive medications that could lead to harm unless treatment was part of randomised controlled clinical trials. Australia called for the allocation of resources to research into factors protecting and aiding recovery from Covid-19 infections in the older population but, in comparison to Brazil, did not speak out against treatment outside the remit of research.

#### Social care

This category includes codes relating to forms of care and support other than or in addition to healthcare. Some societies (e.g. Australia, Ireland) demanded that gaps in health and social care provision must be addressed urgently. One society (Germany) highlighted the need to find innovative approaches to care during the pandemic to ensure high quality care, balanced with protective measures. Various NGGS urged that the pandemic and lessons arising from it be used to improve care and support for older adults. This included calls for future investment in health and social care (UK), reduction in ageism in health and social care (Argentina), and improved long-term support in the community after hospitalisation (Portugal). One society (Ireland) urged that the pandemic be the starting point for a discussion about the future of care for older adults in society.

#### Care at home

Various NGGS endeavoured to promote the same concern for people receiving care at home compared to individuals in institutional settings or hospitals during the pandemic. Some societies (Ireland, Argentina) called for high quality care during and post-pandemic, whether at home or in other care settings. The NGGS in Germany demanded that older adults cared for at home and their relatives be afforded opportunities for social participation.

#### Hospital care

Codes under this sub-category were frequently linked to the sub-categories of institutional care and healthcare. As such, some NGGS advocated for the admission/discharge to and from hospitals of older patients (mostly care-home residents) with Covid-19 infections (Austria, Portugal, Spain) and/or continued access to hospital treatment for older adults requiring healthcare outside a Covid-19 infection (Australia, Germany).

#### Care in the community

Codes relating to “care in the community” and “community” frequently overlapped. Various NGGS (Germany, UK, Czech Republic) demanded the creation or support of community resources during the pandemic and beyond to support older adults. Some (Ireland, Portugal) sought improved community care for older adults, including set-up of clinical support programs. Two societies (UK, Australia) urged support for community-based organisations that help to prevent or combat social isolation and loneliness.

### Interventions

Measures to prevent infections among older adults requiring care and support, whether at home or in acute or institutional care settings, were frequently demanded (Czech Republic, Finland, Argentina). Interventions to provide personal protective equipment (PPE) for carers and diagnostic tests in care settings featured prominently. Furthermore, calls for the development of information, guidance and learning materials for health and social care staff, older adults and family members included demands for guidance on how to stay healthy, how to effectively self-isolate and treat patients suspected of being infected, as well as clear information for patients to enable informed healthcare decision making. Various NGGS requested concrete support initiatives for community-dwelling older adults. This included financial supports for older adults and their carers or for community-based organisations (Canada, UK), designated shopping times for older adults (Argentina) or establishment of emergency contacts to prevent isolation and maintain social participation and wellbeing (Germany).

### Social relations

This category subsumes three themes. First, broad consensus existed among relevant statements, including in the UK and Czech Republic, that positive family relationships must be supported during the pandemic. This included demands for the promotion of communication and contact between older adults in acute or institutional settings and their families, support for families who were caring for older relatives at home, and support for people ageing without families.

The second theme related to building a fair, cohesive society, in which everyone is aware of and acts on their obligations towards others. Ideas around a fair society centred on mutual respect of rights, mutual support, and promoting older adults’ social participation despite physical distancing and isolation policies and advocacy. Third, various CFA related to the pandemic’s social impacts on older adults, demanding a holistic and integrated treatment of health and social ramification of any policies, research and political responses.

### Communication and messaging

This category encompassed two themes. First, there was broad consensus around a responsible public discourse and the power of language in the media either supporting or challenging ageism in society. NGGS appealed to journalists, politicians and medical experts speaking in public to be aware of the potential impact of their words on exacerbating ageism and to choose non-ageist language. Some NGGS proposed neutral wording, including replacing the term “elderly” with “older adults”.

Second, some NGGS appealed for more inclusive messaging and media. Specifically, various statements demanded that information, guidance and policies concerning the pandemic be communicated in accessible, easily understandable ways. Some suggested developing TV and radio programmes targeting older adults. Germany asked for older adults’ voices to be heard on TV and radio to capture their Covid-19 experiences*.*

### Human rights

Commonly, NGGS called for recognition, protection and respect of older adults’ “equal rights” during the pandemic, as either enshrined in international or national law. Codes included references to “human rights”, “rights”, legal rights or “constitutional rights” or concepts linkable to human rights, notably “autonomy”, “equality”, “choice and control” or “self-determination”.

Rather than mentioning rights per se, various CFA referred to rights-grounding values, such as dignity and fairness or autonomy, which according to CFA must be protected in responses to the pandemic. In two cases, there were specific references to particular human rights, notably rights to social participation (Germany) and equal access to healthcare (Portugal). In Germany, the CFA balanced the right to social participation with a need to ensure infection control.

### Care workers

The working conditions and training of people providing health or social care in hospitals or care homes were of shared interest among NGGS. Specifically, there was general consensus that care workers must be afforded safe working conditions during the pandemic, including adequate provision of PPE and access to regular testing for Covid-19, to prevent infection among staff and older adults in acute and/or long-term care settings. Various CFA demanded adequate training for care workers to provide comprehensive care for older adults during and post-pandemic.

### Policy

Many statements urged governments to balance policy responses with potentially negative consequences for older adults. The NGGS in the UK, for example, called for innovative policies to enable older adults to meet loved ones amidst physical distancing measures, facilitate attendance at funerals, and enable physical exercise as matters of overall wellbeing and health. Various statements rejected policies based on chronological age alone, which societies argued would reinforce ageist stereotypes and deny the heterogeneity of older populations. Two societies (Portugal, Ireland) demanded the creation of government entities for older adults to ensure that older people’s needs were acted upon in government both during and post-pandemic.

### Ageism

General consensus existed among relevant NGGS that the pandemic and the associated situation of older adults had exacerbated ageist practices. Particularly, many NGGS identified a blanket approach to the group of “older adults” with a lack of understanding of the heterogeneity among older age groups as a form and source of ageism. As such, NGGS, including in Argentina and Ireland, called for recognition of the diversity of older people. The international campaign “Old Lives Matter”, initiated by France, and some national societies (UK, Portugal) urged a shift in perspective, away from considering older adults a burden towards viewing them as valuable members of society who contribute to the economy, communities and families.

### Health

Various NGGS, including in the UK, Portugal and Canada, called for greater understanding of how multiple economic, socio-structural and social factors may influence health needs and outcomes for older adults in the pandemic including beyond a Covid-19 infection.

CFA of various NGGS (e.g. Germany, France, Finland) focused on the needs and care of older people with multiple or underlying health conditions. The relevant CFA indicated that multimorbidity and underlying health conditions must not be a reason to restrict healthcare, with the society in Germany urging older people with underlying health conditions to continue visiting clinics when necessary, and that in Finland demanding that information on older people’s functional capacity be taken into account when planning treatment after a Covid-19 infection.

## Discussion

Our findings should be interpreted within the context of several limitations. We do not claim to have sampled an exhaustive list of NGGS position statements. The focus on position statements as defined by this study may have led to the exclusion of other types of documents or means which serve the same or similar purposes. Whilst making use of Ayalon et al. (2020) categorisation of topics of interest to answer our research question, we acknowledge that the original categorisation is based on academic publications, with its own limitations (p. 7). However, our approach does allow us to draw on key evidence to identify and discuss NGGS’ priorities during the first wave of Covid-19 infections. The article does not aim to propose research priorities using Ayalon and colleagues’ framework. Rather, we seek to draw out shared interests of NGGS as the pandemic unfolded and to define values from these interests to inform future international collaborations in gerontology.

### Position statements: World regions

Most statements in our sample were produced by European NGGS, with some published by NGGS in other world regions, notably Latin America. African and Asian societies associated themselves with an international statement originating in France. Together, analysis of these statements can indicate shared global interests and values that can inform the discipline of gerontology and future international research collaborations.

### Topics of Interest

Within the context of the public health threat posed by Covid-19, the category of long-term care and formal care with its sub-categories was most widely represented in NGGS statements during the pandemic’s first wave. This reflects the on-average high biological vulnerability of older adults to Covid-19 and the pandemic’s multiple negative consequences on the mental and physical wellbeing of people requiring care and support and their informal carers (Hodgson et al. 2020). Globally, providing health and social care during the pandemic has posed numerous ethical and practical problems. It is, therefore, not surprising that this category featured prominently in this analysis, highlighting a shared importance attributed by NGGS to the topic as the pandemic unfolded.

The next four categories generating most attention in NGGS statements related to provision of targeted interventions to solve problems associated with the pandemic, and issues around social cohesion, quality of communication and messaging, and equality and human rights. The most prominent topics suggest that NGGS continued to show considerable interest in traditional subjects of geriatric inquiry (long-term care and formal care) but also growing recognition of social, cultural and legal topics of gerontological interest. Perhaps surprisingly, comparing our study’s topics of interest with those of Ayalon et al. reveals an under-representation of the area of cognitive functioning and an over-representation of the category of moral, ethics and human rights during the pandemic. Our findings thus depart from what Ayalon et al. (2020) describe as a strong focus on a biomedicalisation of ageing and deficits, cementing gerontology’s central goal of addressing “the problem of aging” (p. 5). Rather, this study’s statements reveal heightened gerontological interest in older adults’ position in society and a forward-looking focus on how gerontology can help to achieve greater age-related equality. This aligns with a reinvigorated debate around an International Convention on the Rights of Older Persons, reflecting Georgantzi’s (2021) argument that gerontological insights into the realities of older adults’ experiences should increasingly inform human rights advocacy, law and policies. However, with dementia identified as one of the main underlying health conditions associated with Covid-19 mortality (Wang et al. 2021) and studies documenting the adverse effects of policies in the pandemic on the wellbeing of people living with dementia and their carers (Giebel et al. 2020), the situation of people with dementia in society as a whole could be considered as a part of this interest in an equitable and inclusive society.

Of the nine categories which remained uncoded among the statements, five (nutrition, suicide, addiction, sleep and immigrants) represent also the least addressed topics in Ayalon et al. (2020) analysis. As argued by Ayalon et al. it is possible that some of these topics, nutrition and sleep specifically, are traditional areas of inquiry in disciplines other than gerontology (p. 6). However, addiction, substance abuse and suicide in older adults who were isolated during the pandemic has been identified as a potential problem (Jemberie et al. 2020; Satre et al. 2020). Some studies also suggest a relatively high psychological resilience among older adults generally, with comparatively less adverse mental health outcomes, including suicidal tendencies, among older than younger adults (Vahia et al. 2020). However, it is yet to be determined whether the relatively high levels of subjective wellbeing have endured over the course of the pandemic (Vahia et al. 2020). Other under-represented categories in NGGS statements, including the situation of immigrants (the category of ethnicity and minorities was also under-represented) or people living in rural rather than urban areas, are also important in the light of available evidence. Carr (2021, p. 2), for example, highlighted a stark racial divide in negative outcomes for health and social welfare related to Covid-19, with black and Latinx older adults in the United States particularly affected. Rivera-Hernandez et al. (2020) reported greater susceptibility to Covid-19 among rural- than urban-dwelling older adults in Mexico. Rather than indicating disinterest, these under-represented topics may have been too specific for NGGS to consider within the timeframe when statements were written. As with cognitive functioning and dementia in particular, these under-represented issues can be considered as important factors for research into inclusivity and equality.

### Agreement and disagreement

Our findings suggest a high level of agreement within the categories across NGGS and no strong divides between countries or world regions. However, notable differences in statements arose, specifically in the category of “institutional care” and the question of hospitalisation or isolation of care-home residents with Covid-19 infections. This topic was salient across numerous countries, with Burton et al. (2020) indicating the need to shield care homes from introduction of the virus and the requirement for rapid action in the case of first infections to minimise causalities. The most efficient way to protect care-home residents was yet to be determined at the time when most statements were published. Ireland’s position on questioning the desirability of institutional care in society was particularly notable, as was Brazil’s statement on medicating people with Covid-19 infections outside clinical trials, and the prominence of references to human rights in Latin American statements. In Ireland, the national context of abuse in institutional settings (e.g. BBC 2021) potentially influenced its position on care homes for older adults. Latin America is the only world region with a dedicated Convention on Protecting the Human Rights of Older Persons, which may explain this topic’s prominence in relevant statements. While these examples highlight the importance of contextualising NGGS statements according to country of origin, such analysis is beyond the scope of this study.

### Overarching themes

Reflecting upon our findings, we identify three overarching themes. A first theme concerns the *advocacy for equality and inclusion* of older adults not only in the immediate pandemic response, but also beyond. Studies show that the pandemic and the contrasting risks to the health of older as compared to younger people without underlying health conditions has led to a global surge of ageism (Ayalon 2020; Aronson, 2020). This includes commonplace use of ageist language, stereotyping and policies either based on ignoring older adults or segregating them from the rest of society (Ayalon et al. 2020). NGGS recognised the surfacing of ageism and discrimination and advocated for equal treatment, non-discrimination and inclusion of older adults and their carers, whether in the community, workplace, media, or health and social care settings.

A second overarching theme is *a holistic approach to care* of older adults with health and/or support needs. The CFA in NGGS statements suggest an approach in which all stages and actors in an individual’s health and social care journey are equally important and should work in an integrated manner for best personal outcomes. Studies highlight challenges to delivering integrated care during the pandemic and opportunities for learning post-pandemic (e.g. Lindner et al. 2020; Stein et al., 2020). Here, NGGS and their members can continue to make a contribution through research and sharing of best practice.

The third overarching theme was the willingness and ability of NGGS to showcase the *academic discipline of gerontology as an applied discipline*, which can directly inform policy making, public communication, health and care systems and other areas to improve the lives of older and younger people. Gerontology’s interdisciplinary nature and its multi-method research approaches mean that gerontologists can address all aspects of age and ageing during and after the pandemic and even adopt advocacy roles at national and international level. Drawing these points together, we conclude that high levels of agreement amongst the world’s professional gerontology and geriatrics societies on priority areas during the first wave of the Covid-19 pandemic points to a shared set of values and visions for older adults and provides a basis for future global research collaborations in gerontology.

## Supplementary Information

Below is the link to the electronic supplementary material.

Supplementary file1 (DOCX 30 kb)

## Data Availability

All data have been uploaded onto a data depository available at: https://tinyurl.com/jjdutr69.

## Code availability

Nvivo.
