# Supplementary material for: Responding to Covid-19: an analysis of position statements of gerontological societies worldwide
Source: Eur J Ageing. 2022 Apr 18;19(4):1229–41. doi: 10.1007/s10433-022-00700-7 (PMC9014281; doi:10.1007/s10433-022-00700-7)
Supplement: Supplementary file 1 — Supplementary file1 (DOCX 30 kb) [file 10433_2022_700_MOESM1_ESM.docx]

**Appendices of supplementary material**

**Supplementary Material A1: Coding framework**

| **Main categories, sub-categories and third level categories** | **Specification of categories according to Ayalon et al. 2020** | **Rulebook and manual per category as used in this study** |
| --- | --- | --- |
| 1. Cognitive functioning | dementia, Alzheimer’s disease, cognitive decline and cognitive functioning | dementia, Alzheimer’s disease, cognitive decline and cognitive functioning |
| 1. Long-term care and formal care | hospital care,  formal care, home care and nursing homes |  |
| 2.a Care settings (sub-category) |  |  |
| 2.a.a Institutional care setting (third level category) |  | care home, nursing home, aged care facility, retirement home |
| 2.a.b Home care setting |  | care at home including extra care and retirement villages |
| 2.a.c Hospital care setting |  | hospital, intensive care, in-patient |
| 2.a.d Care settings in the community |  | GP surgeries, day care centres, resource centres, etc. |
| 2.b Health and social care |  | overarching concepts: care quality, person-centred care, care, care systems/care planning |
| 2.b.a Health care |  | geriatric care/palliative care/geronto-psychiatric/rehabilitation/acute and emergency healthcare/therapeutic care/symptom control/nursing care AND ways of providing healthcare—telecare/consultations/telephone psychiatric care, etc. |
| 2.b.b Social care |  | social care, social care systems, support with needs, needing help of others |
| 1. Emotional and personality | wellbeing,  emotional functioning, coping mechanisms and locus of control |  |
| 1. Health | medical conditions and chronic illnesses | physical health, illness, multimorbidity, long-term conditions, geriatric conditions, geriatric patients, multimorbid patients |
| 1. Family and informal care | family care and informal (unpaid) care | informal care through family, friends and neighbours, informal/family carers |
| 1. Physical functioning | functional ability, disability, activities, people with complex care needs |  |
| 1. Mental Health |  | mental health conditions, depression, anxiety, mental health, mental wellbeing |
| 1. Interventions | treatments, programs, interventions | Intervention/preventive/diagnostic measures, initiative, specific solutions, hygiene measures, physical distancing—institutionalised, diagnostic measures/testing |
| 1. Countries and nations |  | international perspectives, differences between countries (explicitly other countries) |
| 1. United States |  | United States, USA |
| 1. Gender and sex |  | gender, male, female, sex, LGBTQ+ |
| 1. Community |  | community; neighbourhood; community resources; community organisations |
| 1. Work and retirement |  | work, retirement, working conditions, pay |
| 1. Social relations |  | society; social relationships; social cohesion; social isolation; loneliness; social interaction; friends; family; neighbours |
| 1. Communication and messaging |  | media; communication; messaging; rhetoric; language, words, saying/talking; visual images |
| 1. Successful aging |  | protective factors/resources; optimal aging; resilience; adaptation, healthy, quality of life independence, risk factors to aging |
| 1. Ethnicity and minorities |  | ethnicity, minority, BAME |
| 1. Care workers |  | professional care workers; nurses; social care staff; foreign nursing assistants; GPs, health visitors  Issues around staffing, pay, working conditions, training |
| 1. Marital relationship |  | spouse, partner |
| 1. Economic status and poverty |  | economic status, material resources and poverty |
| 1. Policy |  | policy, laws, regulations, action plans followed by a [government](https://dictionary.cambridge.org/de/worterbuch/englisch/government), a [political](https://dictionary.cambridge.org/de/worterbuch/englisch/political) [party](https://dictionary.cambridge.org/de/worterbuch/englisch/party) and other organisations/groups of [people](https://dictionary.cambridge.org/de/worterbuch/englisch/people), social/physical distancing—blanket |
| 1. Ageism |  | ageism and discrimination (sentences that include the words ageism, ageist/racism/sexism or discrimination based on age/gender/race/religion, etc.  Sentences that mention discriminatory practices/use of ageist language/stigmatisation  Sentences that express value judgments about ageist/discriminatory practices) |
| 22.a Heterogeneity/diversity of older population |  | population aging; demographics; characteristics of older people; definition of older age |
| 1. Death |  | deaths, dying, deceased |
| 1. Geriatrics and gerontology research and education | gerontology research, geriatric curriculum |  |
| 1. Rural and urban |  | rural, urban, city, country |
| 1. Life span |  | life span; life course; life cycle |
| 1. Longevity and mortality |  | longevity, mortality |
| 1. Technology |  | telephone, mobile, Internet, messaging services, video call, robotics, AI |
| 1. Religion and spirituality |  | religion, spirituality, religious rituals |
| 1. Elder abuse |  | abuse; violence; neglect; fraud |
| 1. Frailty and vulnerability |  | frailty; vulnerability; frail; vulnerable (explicit) |
| 1. Immigrants |  |  |
| 1. Intergenerational |  | intergenerational relations, intergenerational solidarity, intergenerational relationships,  intergenerational programs |
| 1. Moral, ethics and human rights |  |  |
| 34.a Ethics |  | moral framework, ethics |
| 34.b Human rights |  | human rights law, articles, rights, dignity, respect, choice, autonomy |
| 1. Volunteering | effects and the motivations for volunteering among older adults |  |
| 1. Sleep | sleep habits, insomnia and fatigue |  |
| 1. Addictions | alcohol and drug addiction |  |
| 1. Suicide | suicidal ideation and acts |  |
| 1. Nutrition | nutrition, appetite, feeding, and diet |  |
| 1. Unclassified |  | sentences that cannot be coded under any of the other codes |

**Supplementary Material A2: Exemplary illustration of coding process—step 2 (quantitative content analysis)—using the calls for action in one of the sampled position statements (AAG)**

| **Coding units: Call for action sentences** | **Applicable categories in the coding framework** |
| --- | --- |
| “AAG members are in agreement that responses to the current spread of COVID-19 in Victorian aged care facilities must ensure fundamental human rights are upheld throughout all COVID-19 responses based on principles of equity and autonomy (the right to choose)”. | 2.a.a Institutional care setting  34.b Human rights  21 Policy |
| “This includes: Ensuring aged care facility residents receive the same access to health care (including admission to acute care hospitals) as Australians living in other types of accommodation”. | 2.a.a Institutional care setting  2.b.a Healthcare |
| - “Involving older people, their families and significant others in healthcare decisions, which includes foregrounding older people’s needs, preferences and goals”. | 2.b Health and social care  2.b.a Healthcare  14 Social relations |
| - “Increasing the number of health care clinicians (including registered nurses, nurse practitioners, general practitioners, geriatricians and allied health professionals) available to support residential aged care facility staff”. | 18 Care workers  2.a.a Institutional care |
| - “Providing in-reach acute health services in residential aged care facilities to support older people who want to remain in place during the pandemic, including older people who test positive to COVID-19”. | 2.b.a Healthcare  2.a.a Institutional care |
| - “Increasing the capacity of residential aged care facilities to respond to the current and any future COVID-19 outbreaks through a national coordinated infection control response model”. | 21 Policy  2.a.a Institutional care |
| “Age must be discussed sensitively and respectfully when developing policy responses to Covid-19”. | 15 Communications and messaging  21 Policy |
| “An ethical framework to guide resource rationing must be developed before the point of needing to ration resources is reached”. | 34.a. Ethics  8 Interventions |
| “This must include the voices of older people, including those captured in the Health Issues Centre April 2020 forum report “Who Makes the Call?”. | 15 Communications and messaging |
| “Usual” care needs to be boosted in the face of COVID-19”. | 2.b. Health and social care |

**Supplementary Material A3: Exemplary illustration of coding process—step 3 (qualitative content analysis)—using one of the categories (14 Social relations)**

| **Main category** | **Themes** | **Sub-themes** |
| --- | --- | --- |
| Social relations | Foster family relations | Enable visits; foster family connections; support people in the family; support people without families; allow families at end of life |
|  | Adapt systems | Recall social aspects of health and ageing; adapt health services to social crisis; remember need for social support |
|  | Build a fair society | Reflect on society’s obligation; ensure social interaction and participation; foster social cohesion; prioritise needs of older people; beat social isolation; involve people |

**Note.** This table outlines the coding framework after step 3 in the coding process (qualitative content analysis) was completed. The main category was “social relations”. Coding units (i.e. calls for action sentences in the NGGS’ statements) coded under this category were inductively analysed, from which three themes arose. The coding units under each theme were further inductively analysed for sub-themes.

**Supplementary Material A4: Supporting quotations per category**

| **Category** | **Findings of qualitative content analysis per category** | **Illustrative quotations** |
| --- | --- | --- |
| Institutional care | In main text | “AAG members are in agreement that responses to the current spread of COVID-19 in Victorian aged care facilities must ensure fundamental human rights are upheld throughout all COVID-19 responses based on principles of equity and autonomy (the right to choose). (Australia, Responding to Covid-19 spread in Victorian aged care facilities)” |
|  |  |  |
|  |  | “A general order for complete isolation of care home residents, through prohibition to leave the room or the nursing home grounds… must be rejected. (Germany, Facilitating social participation of older adults despite the Corona pandemic)” |
| Healthcare | In main text | “The care and maintenance of human life is a supreme good, which is to be sought for all people, and which should only give way to end-of-life or palliative care when the advance of the illness or deterioration determine that all necessary measures for comfort and a good death must be taken, with the appropriate care. (COMLAT)” |
|  |  | “Ensuring aged care facility residents receive the same access to health care (including admission to acute care hospitals) as Australians living in other types of accommodation. (Australia, Responding to Covid-19 spread in Victorian aged care facilities)” |
|  |  | “It should therefore be possible to fully and unreservedly provide and settle the options for treatment by video that have already been implemented for old and vulnerable patients in the form of treatment by telephone as well. (Germany, Facilitating telecare for psychotherapy for older and vulnerable patients)” |
| Hospital care | In main text | “The early rehabilitation care of geriatric patients after surviving a COVID 19 infection must be planned promptly in order to relieve the primary care facilities and to enable a prompt return to home care or to a sheltered care facility. (Austria)” |
| Social care | In main text | “The organisations have stated that the COVID-19 pandemic has further highlighted healthcare and support gaps experienced by older people which must be urgently addressed. (Ireland, Call on government to take action to provide long term change)” |
|  |  | “Creative measures are required to support this “care at a distance” by means of new care structures, new communication channels and social services that benefit both the person in need of care as well as the caring relatives. (Germany, Facilitating social participation of older adults despite the Corona pandemic)” |
|  |  | “We have a real opportunity now to reframe and rephrase how we discuss and talk about ageing, look after people and support the ageing population in our country. (Ireland, Mind Your Language)” |
| Care in the community/  community | In main text | “The conversation about what we have learnt, and how we can improve and transform the care that older people receive in our communities, must start now. (Ireland, Call for action for long-term change)”. |
| Interventions | In main text | “To reduce the risk of infection, the visitor should be provided with PPE [Personal Protection Equipment] if the patient in question has coronavirus infection. (Finland)” |
|  |  | “Given the centrality of both mental and physical well-being, there is a need for clear guidance on what people can do to maintain and improve their physical and mental health while keeping physically apart from others. (UK)” |
| Social Relations | In main text | “Special thought should be given to how people might connect with loved ones who live in care settings. (UK)” |
|  |  | “We need to find ways support each other, share knowledge and be kinder to each other. (Portugal)” |
| Communication and messaging | In main text | “We must ensure a more sensitive use of language in the media and medical reports and avoid age-discriminatory statements. (Austria)” |
|  |  | “These mechanisms must ensure that the information provided is adequate, clear and opportune, available on a non-discriminatory basis and accessible, and presented in a way that is understandable in accordance with the cultural identity, educational level and communication needs of the older person. (Argentina, Old age in the pandemic: a matter of rights)” |
|  |  | “This means that older people themselves have a voice in public media and must be addressed as active individuals capable of decision-making and acting. (Germany, Facilitating social participation of older adults despite the Corona pandemic)” |
| Human rights | In main text | “In the current situation, clear and ethical guidance from the national authority is needed to implement quarantine and isolation measures in 24-hour care units in order to avoid violating the fundamental rights of vulnerable people due to varying practices, inadequate guidelines or resource problems.(Finland)” |
|  |  | “We must use the lessons learnt during the first phase of this pandemic as an opportunity to provide a cohesive service for older people and enable them age well, safely and with dignity where they are living. (Ireland, Statement on the Expert Panel Report on Covid 19 and Nursing Homes)” |
|  |  | “The right of people in need of care to social interaction, social contact and good care must be balanced with significantly improved protection against infection. (Germany, Facilitating social participation of older adults despite the Corona pandemic)” |
| Care workers | In main text | “Let’s join together in demanding provision of sufficient effective protective equipment for health professionals, but also for the staff of residential and field social services, and for lay carers. (Czech Republic)” |
|  |  | “People providing the care to those with complex needs must have access to the right training, education, equipment, advice and support to continue improving the care they provide older people with complex care needs. (Ireland, Statement on the Expert Panel Report on Covid 19 and Nursing Homes)” |
| Policy | In main text | “The map of homes with number of residents, medical and nursing staff per reference hospitals needs to be prepared in each community and a resource plan according to territories needs to be established, to be agreed on between the health and social authority. (Spain)” |
|  |  |  |
|  |  | “We need to find a way to allow people to walk or cycle to local shops, to take exercise (for themselves and their pets), and to wave at one another and make social connections while maintaining a safe distance and observing hygiene requirements, without being singled out or intimidated. (UK)” |
| Ageism | In main text | “We must remember that age is just a number, older people are a broad heterogeneous group ranging from very fit to frail, and a one-size solution does not fit all. (Ireland, Statement on the Expert Panel on COVID-19 and Nursing Homes)” |
|  |  | “In 2050, people aged 50 and over will be 2 billion in the world: “society will be able to derive an advantage from this aging population if we all age in better health. But for that, we must eliminate ageist prejudices. (France, “Old Lives Matter”)” |
| Health | In main text | “Raise awareness among the population in general and among professionals dealing with older people and their families on the multiple determiners of health, which are the social aspect, but also economic, legal, planning, habitation, transport, communication, accessibility and new technology. (Portugal)” |
| Frailty and vulnerability | Some NGGS, including SEGG, AAG and SFGG, expressed concern about potential policy implications during the pandemic for vulnerable groups and individuals regarded as being frail. While some (SEGG) sought to ensure that vulnerable groups, such as care home residents, were considered in policy making and protective actions, others (AAG) demanded assurance that vulnerable groups and people identified as being frail were not negatively impacted by policy measures. | “Residential homes are the most vulnerable resource in the face of coronavirus and require our support and urgent protective actions. (Spain)”  “Policies aimed at ending the pandemic must respect and be sensitive to members of vulnerable communities…(Australia, Covid-19: control measures must be equitable and inclusive)” |
|  |  |  |
| Geriatrics and gerontology (research/education) | This category had three themes. 1: Interdisciplinarity of gerontological and geriatric research, in which health, sociological and arts/humanities research must be considered as intertwined to tackle pressing issues emerging from the pandemic. Particularly, the CAG took a lead on this theme, whilst DGGG promoted gerontological and nursing research but without highlighting the interdisciplinarity of gerontology. 2: Gerontological and geriatric training of health and care professionals as well as policymakers. NGGS, like CAG, SPGG and SFGG, expressed the belief that such training can improve the quality of acute care in hospitals, decision making in governments, and serve as a factor in fighting ageism. These societies called for a blanket approach to gerontological and geriatric training of various professionals. 3: Research methods in gerontology and geriatric research during the pandemic and beyond. According to some NGGS such research must include and involve people and wider communities affected by the pandemic, for example through qualitative research methods. One society (SBGG) specifically highlighted the need to follow clinical research that has been granted ethical approval to avoid harm to patients involved in medical trials. | “We strongly encourage the adoption of interdisciplinary approaches in the response to COVID-19 because of the value added when connections between and across disciplines are made. (Canada)” |
|  |  |  |
| Family and informal care | Some of the NGGS acknowledged the potentially complex situation and various challenges for family and informal carers of older adults during the pandemic, demanding acknowledgement and support for such individuals. Together, the various CFA form a catalogue of support measures and rights for informal carers. This includes PPE for informal carers in outpatient and institutional care settings (CGGS, DGGG), rights to work from home or compensation for avoiding public transport in the name of risk control (DGGG). | “…health and social care workers, family and friends who will need to provide care to people who become unwell form the front line of society’s response to the pandemic…will need to be acknowledged and treated as such. (UK)” |
| Successful aging | Some NGGS saw a need to foster key components of successful aging during and after the pandemic, including independence, dignity, healthy and active aging and participation. | “Exercise, personal mobility and human contact are key to healthy ageing and need to be promoted long beyond the current pandemic. (UK)” |
|  |  | “We must use the lessons learnt during the first phase of this pandemic as an opportunity to provide a cohesive service for older people and enable them age well, safely and with dignity where they are living.(Ireland, Statement on the Expert Panel Report on Covid 19 and Nursing Homes)” |
| Economic status and poverty | Various codes related to the situation of people in financially precarious situations. Some NGGS (BSG, CAG) demanded research into the effects of the pandemic on people from financially disadvantaged backgrounds. Another society (DGGG) called for financial support for family carers and resources for older adults experiencing financial difficulty. | “The research community will need to meaningfully engage with groups who are facing this pandemic from a precarious position.  (Canada)”  “Care services, such as municipal services, health care services and food provisioning services, that older people want or need to utilise due to their current psychological, physical or socio-economic situation, must therefore also be made accessible during the coronavirus pandemic whilst conforming to protective measures. (Germany, Facilitating social participation of older adults despite the Corona pandemic)” |
|  |  |  |
| Mental health/Emotionality and personality | Some NGGS recognised the potentially detrimental effects of policy responses to the pandemic on the mental health and emotional wellbeing (DGGG, BSG, CAG) of older adults and their relatives. They called for relevant research on measuring psychological effects and designing solutions, such as dealing with mental health disorders (DGGG) and grief (BSG)*.* | “The psychological and social effects of the COVID-19 pandemic and the accompanying measures on the situation of people in need of care and their relatives must also be continuously examined, assessed and taken into account when designing further measures. (Germany, Participation and care for people in need for care in times of Corona and beyond)” |
| Moral and ethics | A few NGGS asked for the adoption of an ethical framework for rationing resources rather than group-based policies, for example by age. One society (BSG) demanded that moral judgments regarding clinical decisions be made on need rather than chronological age. | “All clinical decisions for access to testing and treatment as they unfold should be made on clinical need; using age alone as a criterion for decision making is fundamentally wrong. (UK)” |
| Physical functioning | Some NGGS recognised the potentially detrimental effects of the pandemic and related policy responses on the physical functioning of older adults. As such, two societies (BSG, SPGG) highlighted the importance of promoting mobility as part of the pandemic response. In order to ensure the continuance of support for people with disabilities, some NGGS called for social services to stay open during the pandemic. | “…boosting primary health care, disability, aged care and social services in the face of Covid-19 will ensure that peoples’ usual health care needs are addressed. (Australia, Responding to Covid-19 spread in Victorian aged care facilities)” |
| Cognitive functioning | Some NGGS (DGGG, CAG) highlighted the pandemic’s potentially negative effects on older people’s cognition. A few (ÖGGG, DGGG) referred to the specific situation of people living with dementia in care homes or the community and demanded targeted action. Others argued that greater consideration should be given to dementia-sensitive care concepts (DGGG, ÖGGG). | “Greater consideration should… be given to dementia-sensitive care concepts and adequate forms of communication with people with dementia in everyday nursing care, particularly in the current crisis situation. (Germany, Facilitating social participation of older adults despite the Corona pandemic)”. |
| Work/Retirement and volunteering | Relevant codes overlapped in that they focused on conditions at work and volunteering. The DGGG, for example, urged employers to implement hygiene measures to contribute to safe working environments for all employees, regardless of age, rather than asking older employees to stay at home. Others (CGGS, SPGG) demanded that older adults should be able to engage in volunteering activities during the pandemic and promoted “anti-ageism” training of volunteers beyond the pandemic. | “If… people, regardless of age, wish to get involved in voluntary work, let’s not degrade them by rejecting them for “being too old. (Czech Republic)” |
| Technology | Some NGGS (DGGG, BSG, IGS) highlighted the potential of technology to aid social connection during and after the pandemic and enable older adults to age at home. Specifically, they demanded action towards bridging digital divides (BSG) and ensuring access to technology and the Internet in care homes (DGGG). | “We should be thinking of this period as an opportunity to bring people and generations together, especially by helping to bridge digital divides across society where these exist. (UK)” |
| Elder abuse | Some societies (AAG, SAGG, CAG) drew attention to increased and new cases of elder abuse during the pandemic, because of overstretched carers and older adults’ isolation. They thus demanded action to monitor and prevent abuse of older adults during the pandemic. | “We must be alert and prevent situations of physical or psychological abuse by carers who are overstretched and/or stressed by the imposition of quarantine. (Argentina, Old age in the pandemic: a matter of rights)” |
| Countries and nations | The CAG suggested the value of countries learning from others’ policy and public health responses to the pandemic*.* Other NGGS highlighted the importance of particular concepts and regulations proposed by the World Health Organization to shape policy responses internationally, notably that of active and healthy aging (SPGG) and no medical treatment for Covid-19 outside formal medical research (SBGG). | “It is imperative that we take advantage of the “natural experiment” presented by differing policy and public health responses taking place in the multiple “laboratories” of different jurisdictions. (Canada)” |
| Longevity and mortality/death | A few NGGS called for scientific investigations into mortality rates during the pandemic, particularly in regard of the relationship between inequality and mortality (BSG). SPGG demanded policies during and beyond the pandemic for the quality of life for older adults that also promote the valuing of longevity as an essential part of battling ageism in society. | “In this unprecedented period, we call for urgent and ongoing data collection and rigorous analysis of social and economic inequalities, and of the impact of inequalities through this crisis on the living conditions of people, their mental and physical health, and mortality.(UK)” |
| Ethnicity and minorities | The CAG demanded targeted research focusing on the impact of Covid-19 on older adults from ethnic minorities, recognising that the pandemic might affect differently older adults from diverse ethnic backgrounds. | “To understand the impact of COVID-19 on older visible/ethnocultural minorities, a range of methodological approaches and culturally appropriate techniques will be required. (Canada)” |
| Intergenerational/Life span | Some NGGS saw a threat as well as an opportunity in the pandemic regarding intergenerational cohesion. This was a less common category but with CFA from SPGG and IGS both directly linking the concept of “lifespans” and “aging” to the issue of tackling ageism. Whilst SPGG suggested the need for anti-ageist work among people of all ages, IGS advocated for an understanding of anti-ageist work that is not only interested in the non-discrimination of currently older adults but considers it an investment into every person on their aging journey. | “While we need to recognise the important role all these people play in our lives and society today, we must also understand that we need to be better advocates for the requirements of all of us as we age. (Ireland, Mind Your Language)”  We need to find a way of living more comfortably with the increased average lifespan and not rejecting our ageing population… the Portuguese Society of Geriatrics and Gerontology presents all these reflections and proposals as part of a Training and Valuing of Age throughout the Whole of Life. (Portugal)” |
|  |  |  |
